# What Sport Activity Levels Are Achieved in Patients After Resection and Endoprosthetic Reconstruction for a Proximal Femur Bone Sarcoma?

**DOI:** 10.1007/s11999-016-4790-7

**Published:** 2016-03-28

**Authors:** Gerhard M. Hobusch, Jakob Bollmann, Stephan E. Puchner, Nikolaus W. Lang, Jochen G. Hofstaetter, Philipp T. Funovics, Reinhard Windhager

**Affiliations:** 1 Department of Orthopaedic Surgery, Medical University of Vienna, Waehringer Guertel 18–20, 1090 Vienna, Austria; 2 Department of Traumatology, Medical University of Vienna, Vienna, Austria; 3 Orthopaedic Hospital Speising Vienna, Vienna, Austria

## Abstract

**Background:**

Limited information is available about sports activities of survivors after resection and reconstruction of primary malignant bone tumors with megaprostheses. Because patients often ask what activities are possible after treatment, objective knowledge about sports activities is needed to help assess the risks of sports participation and to help guide patients’ expectations.

**Questions/purposes:**

The aims of this study were to evaluate (1) what proportion of patients with proximal-femoral megaprostheses placed as part of tumor reconstructions can perform sports; (2) what activity levels they achieved; and (3) whether sports activity levels are associated with an increased likelihood of revision.

**Methods:**

This retrospective study considered all 27 living patients in our institutional tumor registry with enduring proximal-femoral reconstructions performed more than 5 years ago who were between the ages of 11 and 49 years at the time of the reconstruction; seven were lost to followup and one was excluded because of paraplegia as a result of a car accident and another because of senile dementia; another two were excluded from statistics because of growing prostheses and skeletal immaturity at the time of followup, leaving 16 (11 male, five female) for analysis. Their mean age was 26 ± 12 years (range, 11–49 years) at surgery, and the mean followup was 18 ± 7 years (range, 5–27 years). Types of sports, frequency per week, duration of each sports session as well as the UCLA and modified Weighted Activity Score were assessed retrospectively by an independent assessor a median of 18 years (range, 5.3–27 years) after surgery.

**Results:**

Patients recalled that preoperatively 14 were practicing sports 5 (± 4) hours/week. At followup, 11 of the patients were practicing one or more sports activities 2 (± 3) hours/week on a regular basis. The preoperative UCLA and modified Weighted Activity Score levels of 9 and 6 fell to levels of 6 (p = 0.005) and 3 (p = 0.025), respectively, at followup. With the numbers of patients available for study, we could not determine that prosthetic failures were associated with sport activity levels.

**Conclusions:**

Patients who survive primary malignant bone tumors in the proximal femur reconstructed by megaprostheses are able to perform some sports activities. The estimates of activity levels made in this study probably are best-case estimates, given that some patients were lost to followup; patients unaccounted for might not be doing as well as those represented here. Also, the degree to which sports participation influences implant durability remains, for the most part, unanswered; studies with more patients and longer followup will be needed to determine to what degree prosthesis survivorship relates to sporting activity levels. Most patients perform low-impact sports and at a lower level than they had preoperatively. Because this is a preliminary study of a select group of patients, further information is necessary to weight the benefits of higher sports activity levels against potential risks. If this can be confirmed in a larger number of patients, the information may guide surgeons in their discussion with patients preoperatively and give them some objective assessment of what to expect regarding sports activities.

**Level of Evidence:**

Level IV, therapeutic study.

## Introduction

Current multimodality treatment of primary malignant bone sarcomas has improved patient survivorship to 70% to 80% in the long term. The proximal femur meta- and diaphysis are affected by a variety of primary bone sarcomas and these are relatively common skeletal sites for certain sarcomas [6, 14, 26, 32]. Proximal femur megaprostheses are generally preferred in reconstruction after resection of these tumors with 10-year implant survival rates of 47% to 82% and good function (Musculoskeletal Tumor Society 67%–77%) [32]. Apart from obvious benefits of this treatment, long-term survivors of bone sarcoma may not remain physically active, which could result in some patients acquiring a psychosocial deficit from inactivity [3].

Sport is a leisure time physical activity and has been shown to have a wide range of health benefits [8]. Sports and exercise in patients with cancer may improve fitness and psychosocial health and be of benefit in cancer rehabilitation [29, 30]. Furthermore, sports can enhance a person’s sense of well-being, which may be essential for postcancer patients overcoming this life-threatening event and treatment-associated side effects [1, 8, 24, 28, 29, 31, 33].

Studies in elective hip replacement for arthrosis have shown that patients return to moderate sports activity [2] and can carry out high levels if they were proficient in sport before the operation [11]. Over the years, advances in the design and materials used in hip arthroplasty have improved the longevity of prostheses allowing patients to function for longer periods without revision [7]. However, patients with femur megaprostheses differ from patients after elective joint replacements because of the use of larger, more complex implants, a more invasive operation with resection of bone and soft tissues, and the generally younger age at implantation compared with patients with hip arthritis. Unfortunately, very few data exist with regard to the sports activity levels of patients with megaprostheses after sarcoma resection [15, 16, 18]. Some patients with sarcoma may want to have information about activity after treatment, but little objective information exists in general [22] and even less concerning postoperative sport activities in patients who have been treated with a proximal femur megaprosthesis for a malignant bone tumor.

Hence, the aims of this study were to evaluate (1) what proportion of patients with proximal-femoral megaprostheses placed as part of tumor reconstructions can perform sports; (2) what activity levels they achieved; and (3) whether sports activity levels are associated with an increased likelihood of revision.

## Patients and Methods

The study was carried out according to the Helsinki criteria and after assessment by the local ethics commission (EK No. 1691/2014).

Inclusion criteria for this study were (1) primary malignant tumor of the proximal femur and reconstruction with a proximal femur megaprosthesis in patients who had a 5-year minimum followup; (2) skeletal maturity at the time of followup; and (3) German-speaking (Austria, Germany, Switzerland). The initial decision-making for all resections and megaprosthetic replacements was based on primarily two conditions. The tumor resections had to be aimed at wide margins, for a good oncologic response, and the limb function should at least not be compromised by nerve damage. Every tumor was diagnosed at the local institute of pathology. The basis for the patient selection was the local bone and soft tissue tumor registry from January 1979 until June 2010.

Eighty-seven patients with a primary malignant bone tumor in the proximal femur were treated with proximal femur megaprostheses within this time. Of these 87 patients, 53 patients had died of disease and 34 were thought to be alive at the time of the data collection. Three were not German-speaking and were living abroad. Four were already amputated, one patient had paraplegia as a result of a car accident, and one had senile dementia. These were excluded. Two patients received a growing prosthesis and were skeletally immature at the time of latest followup and were excluded from statistics. However, we found it important to report their demographics (Table 1), sports activity, and complications (Table 2). Seven patients were unavailable and lost to followup. Overall there were 16 (11 male, five female) patients (18% of the initially operated) with a mean age of 26 ± 12 years (range, 11–49 years) at surgery and a mean followup of 18 ± 7 years (range, 5–27 years) included in this study (Table 1).

**Table 1:** Demographics of long-term survivors after malignant bone tumors after resection and reconstruction with proximal femur megaprostheses*

Patient number	Gender		Followup (years)	Type of prosthesis	Cup	Trochanter fixation	Tumor	Chemotherapy
1	M	20	18	HMRS^®^	Bipolar head	Suture	Ewing‘s sarcoma	CESS 92
2	M	33	19	HMRS	Bipolar head	Suture	Hemangioendothelioma	CESS 92
3	F	18	18	HMRS	Screw pan	ETA^®^	PNET	CESS 91
4	F	30	25	HMRS	Screw pan	Suture	Fibrosarcoma	/
5	M	23	20	HMRS	Bipolar head	ETA^®^	Osteosarcoma	COSS 86
6	F	25	20	HMRS	Bipolar head	Suture	Ewing‘s sarcoma	CESS 91
7	M	26	10	HMRS	Pedestal cup	ETA^®^	Chondrosarcoma	/
8	M	11	7	GMRS^®^	Bipolar head	Suture	Osteosarcoma	EURAMOS 1
9	M	27	21	HMRS	Bipolar head	Suture	Osteosarcoma	COSS 86
10	M	26	12	HMRS	Bipolar head	Suture	Ewing‘s sarcoma	Euro-Ewing 99
11	F	36	21	HMRS	Bipolar head	Suture	Lymphoma	CHOP
12	M	17	5	GMRS	Bipolar head	LARS^®^	Ewing‘s sarcoma	Euro-Ewing 99
13	M	49	10	MUTARS^®^	Tripolar head	Suture	Myelosarcoma	/CTH n.o.s.
14	F	44	18	HMRS	Bipolar head	ETA^®^	Chondrosarcoma	/
15	M	46	27	HMRS	Bipolar head	Fascia lata	Clearcellsarcoma	/
16	M	47	7	GMRS	Bipolar head	LARS^®^	Chondrosarcoma	/
	Growing protheses							
17	M		7	HMRS*	Screw pan	Suture	Ewing‘s sarcoma	Euro-Ewing 99
18	M		12	HMRS*	Screw pan	Suture	Ewing‘s sarcoma	Euro-Ewing 99

* Included at the bottom of the table are two patients with growing prostheses, who were excluded from statistics because of immature skeletal status at time of followup and their different surgical histories (Table 2); HMRS (Howmedica Modular Resection System; Stryker, Mahwah, NJ, USA), GMRS (Global Modular Resection System; Stryker, Mahwah, NJ, USA), MUTARS^®^ (Modular Universal Tumor And Revision System, Implantcast GmbH, Buxtehude, Germany), sutures for trochanter fixation of the musculature/tendon to the prosthesis, ETA^®^ Enhanced Tendon Attachment (Howmedica Modular Resection System; Stryker); Ligament Advanced Reinforcement System (LARS^®^, Arc sur Lille, France); CTH n.o.s. (chemotherapy, not otherwise specified); M = male; F = female.

**Table 2:** Prosthetic failures and long-term followup sports activities of long-term survivors after malignant bone tumors after resection and reconstruction with proximal femur megaprostheses

Patient number	Gender	Followup (years)	Failures					Service		Metastases	UCLA activity score*	Modified Weighted Score*
			Type 1	Type 2	Type 3	Type 4	Type 5	Service	Lengthening			
1	M	18	/	/	/	/	/	/	/	/	3	0
2	M	19	/	/	2	/	/	/	/	/	8	9
3	F	18	1	1	/	/	/	/	/	/	7	10
4	F	25	2	/	/	/	/	/	/	/	2	0
5	M	20	1	/	1	/	/	/	/	/	6	4,5
6	F	20	1	/	/	/	/	/	/	/	6	7
7	M	10	/	/	/	1	/	/	/	/	7	4
8	M	7	/	/	/	/	/	/	/	/	9	10
9	M	21	/	/	/	/	/	/	/	/	6	4,5
10	M	12	/	/	/	/	/	/	/	/	5	0,5
11	F	21	/	/	1	/	/	/	/	/	7	10,5
12	M	5	/	/	/	/	/	/	/	/	4	0
13	M	10	/	/	/	2	/	/	/	/	3	0
14	F	18	2	/	/	/	/	/	/	/	6	3
15	M	27	/	/	/	/	/	/	/	/	8	3
16	M	7	/	/	/	/	/	/	/	Pulmonary	4	0
	Growing prostheses											
17	M	7	/	/	/	/	/	3	6	/	4	1
18	M	12	/	/	/	/	/	3	5	/	7	32

* UCLA and modified weighted activity scores of latest followup: Failure Type 1 = soft tissue failure, Failure Type 2 = aseptic loosening, Failure Type 3 = structural failure, Failure Type 4 = infection, Failure Type 5 = local recurrence; M = male; F = female.

Five patients had a histological result of Ewing’s sarcoma/primitive neuroectodermal tumor, three patients of osteosarcoma, three of a chondrosarcoma, one of a fibrosarcoma, one of a lymphoma, one patient of a hemangiothelioma, one of myelosarcoma, and one of a clear cell sarcoma. Patients with Ewing’s sarcoma were partly presented in previous work with different tumor localizations [15].

Chemotherapy protocols used for Ewing’s sarcoma included CESS 91 and 92 [27] and Euro-Ewing 99 [17]; protocols for osteosarcoma included EURAMOS I/COSS 86 [34] and for lymphoma CHOP [5]. No definite heart or kidney disease had been diagnosed in these patients over the complete followup.

Resection was carried out according to tumor principles established by Enneking et al. [9]. All patients were treated by cementless proximal femur megaprostheses. Twelve patients received an HMRS proximal femur prosthesis implant (Howmedica Modular Resection System; Stryker, Mahwah, NJ, USA); three patients received a GMRS proximal femur prosthesis implant (Global Modular Resection System; Stryker). One received a MUTARS^®^ silver proximal femur replacement (Modular Universal Tumor And Revision System; Implantcast GmbH, Buxtehude, Germany) secondarily after implant infection. The abductor muscle or trochanter fixation was either done by nonresorbable sutures of the musculature to the prosthesis or with mechanical trochanter fixation using an Enhanced Tendon Attachment (ETA^®^ Howmedica Modular Resection System; Stryker), Ligament Advanced Reinforcement System (LARS^®^, Arc sur Lille, France), or both.

Complications were described by the comprehensive ISOLS failure mode classification including oncologic as well as nononcologic failures as follows: soft tissue failure (Type 1), aseptic loosening (Type 2), structural failure (Type 3) infection (Type 4), and recurrence (Type 5) [13]. In three patients (No. 3, 5, 6), flexion contractions were treated by Judet’s quadricepsplasty; one of the patients (No. 4) developed superficial tissue necrosis that was treated by débridement and meshed skin graft (Table 2). One patient (No. 14) received vessel grafts secondarily after primary vessel resection and development of thrombosis, and one patient had two prosthesis dislocations (failure Type I). One patient (No. 3) had developed cup loosening and was replaced (failure Type II). Another patient (No. 2) had mechanical disconnection of a module and loosening of the cone at another time. One patient (No. 5) with gluteal insufficiency was treated by a trochanteric ETA^®^ attachment; another patient (No. 11) had screw breakage with consecutive prosthesis replacement (failure Type III). Two patients (No. 7, 13) developed prosthetic infection and had two-stage revisions of the prostheses (failure Type IV). One patient (No. 16) developed 13 pulmonary metastases that were resected until 2.5 years before latest followup.

In addition to prosthetic failures, we added patients after implantation of growing prostheses. They needed five and six extension operations. In addition, each of them needed three service operations, which are defined as follows. In both patients with an expandable prosthesis, a Salter pelvic osteotomy and secondary removal of Kirschner wire was performed to treat dislocation of the head. One patient received an acetabular cup and a second patient had the implantation of the growing module secondarily after the primary implantation of a HMRS prosthesis.

Assessment of sport scores was based on a questionnaire-guided recall telephone interview for different time points as described by Lang et al. by independent assessors [18]. The questionnaire was administered at a median of 18 years (range, 5.3–27 years) after the index surgical procedure.

The UCLA score surveys the general level of activity. It ranges from 1 to 10.

A UCLA activity score of 1 means total inactivity, dependence on others, or the inability to leave your residence; a UCLA activity score of 10 corresponds to regular participation in contact sports [19, 24, 25]. UCLA seems to be the most appropriate scale for assessment of physical activity levels in patients undergoing total joint arthroplasty [23].

The modified Weighted Activity Score has to be calculated. It collects the sport performance on different levels. It is calculated by taking the impact factor of a sport with the number of sport sessions per week as well as the duration of the sport sessions and multiplying it into hours. The impact factor of a sport session complies with the assessment by the Knee and Hip Society 2005 [20, 21].

### Statistical Analysis

For metric variables as well as the outcome parameter, the number of valid data, the median, minimum, and maximum were used, respectively. To compare the sports scores and frequencies of different time points, differences were calculated by the Wilcoxon test for paired random samples. To compare gender, different megaprostheses, and trochanteric fixations and complications, the Mann-Whitney U test was used. For the correlations of the relative and absolute prosthesis length, the rank correlation coefficient according to Spearman was calculated. A p value of p < 0.05 was accepted as statistically significant.

## Results

**Fig. 1:**
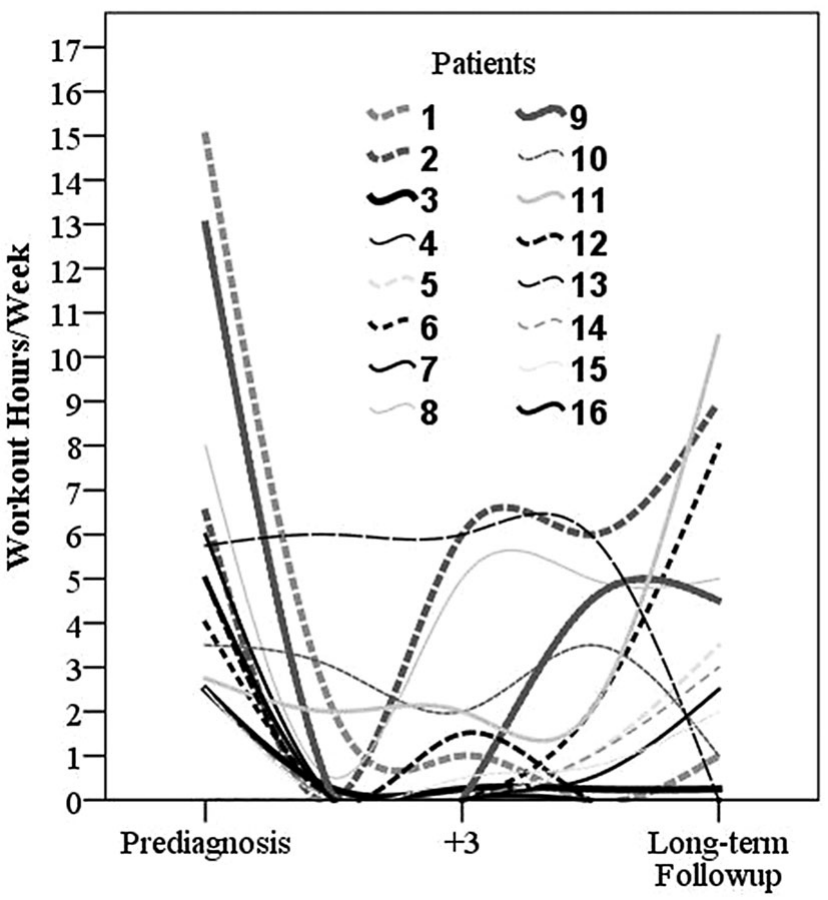
Interpolated line graphs show the workout over the time recalling hours per week of sports activity from before surgery until 5 years postoperatively as well as the actual time point of the latest followup for each survivor after proximal femur reconstruction with megaprostheses. Each line represents a certain patient also described in Tables 1 and 2. Four hours per week represent common recommendations [10] for healthy adults.

Before the operation, 14 of 16 of the patients recalled that they were practicing sport. One year postoperatively, there were six of 16; 3 years postoperatively 10 of 16; and 5 years postoperatively, there were 13 of 16 who practiced sport regularly. The most popular sports before surgery were bicycling in six, alpine skiing in five, jogging in three, swimming in three, hiking/Nordic walking in three, soccer in two, and fitness center in two patients. Five patients were participating in high-impact sports such as jogging, soccer, volleyball, and martial arts. No patient was doing high-impact sports more than 1 year after their operation. At the median latest followup (18 years; range, 5.3–27 years), the most common sports were hiking/Nordic walking in five, cycling/exercise biking in four, swimming in three, and fitness training (fitness center) in three patients. Furthermore, the median hours per week of the sports performed decreased from 4.5 preoperatively until zero 1 year postoperatively (95% confidence interval, 0.0–0.17; p < 0.0001); however, there was no difference with the numbers available from 1 year preoperatively until latest followup (Fig. 1). Cycling and swimming were the only activities continuously performed with an increasing number of patients and increasing workout hours postoperatively. More patients performed Nordic walking/hiking after 5 years postoperatively with increased workout hours. Patients were performing fewer workout hours in the fitness center. Interestingly, one patient started to perform golf postoperatively, although the patient was not participating in golf preoperatively (Table 3). With numbers available, neither (relative or absolute) size of the megaprostheses nor different approaches to trochanter fixation was associated with sport activity.

**Table 3:** Patients’ workout in the course of time in hours/week

Impact	Sports		1 year preoperatively	1 year postoperatively	3 years postoperatively	5 years postoperatively	Long-term followup
			Mean (range)	Mean (range)	Mean (range)	Mean (range)	Mean (range)
Low-impact sports	Cycling	h/w	3 (1–6)	1 (1–1)	2 (1–4.5)	1.8 (0.5–4.5)	2.9 (1–7.5)
		n	6 (38%)	2 (13%)	4 (25%)	5 (31%)	4 (25%)
	Swimming	h/w	0.8 (0.5–1)	1.3 (1–2)	1 (1–1)	1 (1–1)	0.8 (0.5–1)
		n	3 (19%)	3 (19%)	3 (19%)	2 (13%)	3 (19%)
	Hiking/Nordic walking	h/w	2.4 (1.5–3.8)	4 (4–4)	4 (4–4)	2.4 (1–4.5)	5.2 (2–10.5)
		n	3 (19%)	1 (6%)	1 (6%)	5 (31%)	5 (31%)
	Fitness center	h/w	4 (2–6)	2 (2–2)	3.5 (2–5)	3.5 (2–5)	2.5 (1–5)
		n	2 (13%)	1 (6%)	2 (13%)	2 (13%)	3 (19%)
	Golf	h/w			1.5 (1.5–1.5)	1.5 (1.5–1.5)	1.5 (1.5–1.5)
		n			1 (6%)	1 (6%)	1 (6%)
	Alpine skiing	d/y	13 (6–20)	6 (6–6)	8 (6–10)	10 (6–14)	8 (6–10)
		n	4 (29%)	2 (13%)	2 (13%)	2 (13%)	2 (13%)
	Badminton	h/w	2 (2–2)				
		n	1 (6%)				
	Tennis	h/w	6 (6–6)				
		n	1 (6%)				
High-impact sports	Jogging	h/w	1.7 (1–2)	1 (1–1)			
		n	3 (19%)	1 (6%)			
	Soccer	h/w	5 (2–8)	0.5 (0.5–0.5)			
		n	2 (13%)	1 (6%)			
	Volleyball	h/w	8 (1–15)				
		n	2 (13%)				
	Combat sport	h/w	1 (1–1)				
		n	1 (6%)				

h/w = hours per week; d/y = days per year; n = number of patients.

**Fig. 2A–B:**
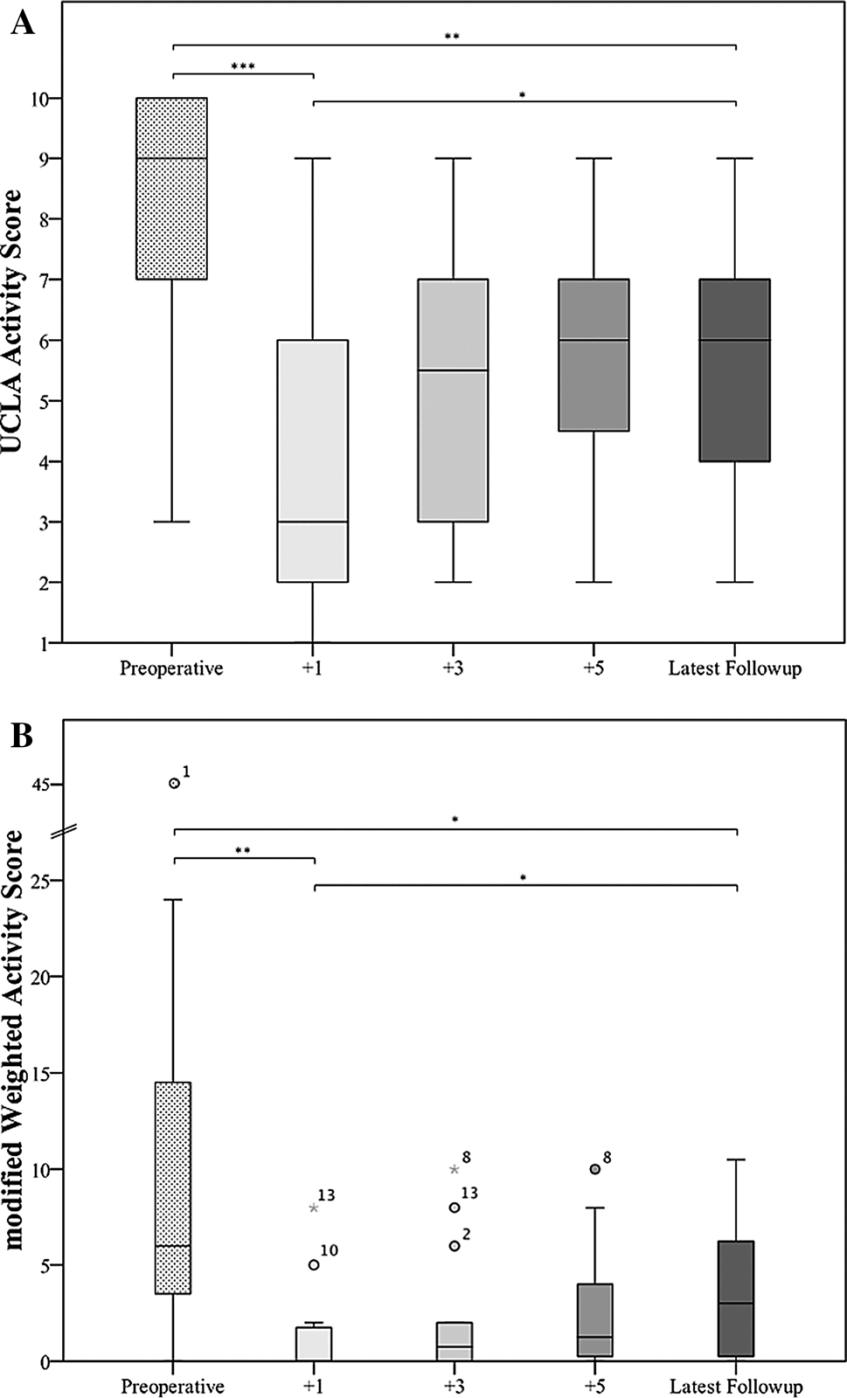
The median UCLA score (**A**) and modified Weighted Activity Score (**B**) for survivors with resection and megaprosthetic reconstruction of the proximal femur are shown preoperatively and postoperatively. Asterisks represent significance (*p < 0.05, **p < 0.001, ***p < 0.0001) in differences of these different time points of this scores.

Most patients achieved their maximum activity levels 5 years postsurgery. The median UCLA activity score fell from a preoperative value of 9 to a long-term followup value of 6 (p = 0.005). The median modified Weighted Activity Score fell from a preoperative value of 6 to a long-term followup value of 3 (p = 0.025). The median UCLA score showed a 3 ([1–9]/9[3–10]) reduction (p = 0.001) after 1 year postoperatively when compared with levels before surgery. When compared with 1-year postoperative levels, there was an increase (5.5[2–9]/3 [1–9]; p = 0.002) until 3 years postoperatively and then it stabilized until latest followup (Fig. 2A). The median modified Weighted Activity Score showed a 100% (0 [0–8]/6 [0–45]) reduction (p = 0.002) at 1 year postoperatively when compared with levels before surgery. With the numbers we had, we could not document an improvement in median modified Weighted Activity Score with further followup (3 [0–10.5]/0 [0–8]; p = 0.092) (Fig. 2B).

Nine of 16 patients had revision of their prosthesis for complications that occurred during the postoperative period. There were no periprosthetic fractures or injuries of the lower extremity bones and joints observed that we could directly relate to the need for revision. However, we cannot exclude a possible relationship of high activity and certain failures in two patients. One developed cup loosening (failure Type II); the other had mechanical disconnection of a module and loosening of the cone at another time (failure Type III) (Table 2).

## Discussion

With improvements in endoprosthesis design and materials and established principles of resection, limb salvage surgery is currently a common method to treat patients with sarcomas of the proximal femur [9]. The main goal for treatment of malignant tumors of the extremities is to maximize survival while preserving a functional limb when at all possible. However, recommendations of permanent limits of sports activities to lessen long-term prosthetic failures by reconstructive surgeons are variable from one surgeon to another [16]. Whereas many patients after elective joint replacement of the hip can still carry out sport activities, some tumor surgeons are reluctant to allow patients with these more complex reconstructions to participate in many athletic activities. Furthermore, activities of daily living, functional outcome, general health, and well-being may be compromised in survivors of bone sarcomas [3]. Some studies have demonstrated that sports activities have potential physical and mental benefits, which may be useful for rehabilitation in patients after bone sarcoma and may reduce the personal burdens of long-term survivors [30, 33]. This case series was selected to look at a specific anatomic site, the proximal femur, to provide information about sport activity levels and prosthetic failures in long-term survivors after proximal femur megaprostheses.

There are several limitations to this study. Most importantly, this study was small, and more than one-fourth of our patients (seven of 27 otherwise eligible patients) were unavailable for followup. The estimates of sports participation made in this study probably are best-case estimates, given that some patients were lost to followup; patients unaccounted for might not be doing as well as those represented here. As such, the degree to which sports participation influences implant durability remains, for the most part, unanswered; studies with more patients and longer followup will be needed to determine to what degree prosthesis survivorship relates to sporting activity levels. Our data were assessed by recall interview at a median of 18 years after the surgical intervention, which may have introduced recall bias regarding sports activities before diagnosis and at different followup time points. However, it is important to note that these patients seemed to accurately recall when they started to perform sports and at what level, indicating the importance of sports for these patients. As a result of the small size of our study population, several variables including gender and technical (trochanteric fixations, cups) and oncological (chemotherapy) issues could not be considered in a multivariable model. It is possible that patients downplayed their sports activities as a result of current recommendations by their surgeons, which were not standardized, and to perform primarily low-impact activities at our department because of the importance of sports activity in the life of a young person. Moreover, these current results, considering types of sports and workout times, might differ between countries and other cultural backgrounds. Our sample size is too small to give general recommendations with regard to sport activities; however, these data may provide information for future studies about sports activities and failures in survivors of malignant bone tumors. It is difficult to generalize these findings to other patients because of differences in the amount of muscle resected, variations in the type of endoprosthesis, and the overall health of the patients. We had a broad age range and sports information from a young person may differ substantially from an older person. We also have insufficient information about the desire of patients to do sporting activities; the ones who did not participate in sports may not have desired to do so rather than being restricted from sports because of complications or recommendations. Also, the findings in this study of patients from Austria may not be reflective of sports activities or desires of patients from other countries or cultures. Furthermore, during this long period between 1979 and 2010, some things could have changed, foremost the advice that was given to patients with regard to sport activity level based on surgeon experiences. Outcomes may reflect the physicians’ advice or beliefs rather than the actual ability. However, according to the principles of limb salvage, the advice the surgeons gave to their patients at our institution was that the limb “shall be moved,” and they have been encouraging them ever since to do moderate sports; they are also reminded that high-impact sports will not be possible, unfortunately without scientific background. However, there was not at all a correlation between followup period and sports activity levels; neither was there a difference between patients’ sports activity and different followups, reflecting that no time-related factors (eg, different techniques) might be involved in the followup of these patients.

This study showed that a high proportion of long-term survivors were active in sports activities. Interestingly, the patients included in this study appeared to be practicing sports that have been recommended after hip arthroplasty for arthritis in larger studies such as general walking (hiking), cycling or ergometer cycling, and swimming (crawl or paddling movements) [4, 35]. One patient started practicing golf during followup and two patients were continuously performing alpine skiing at the latest followup. These two sports are also recommended by hip surgeons, the latter primarily if the patient is already experienced in alpine skiing before surgery [12]. In comparison to the patients in this study, in a prior group of patients we studied with knee megaendoprostheses, sports activity before surgery did not seem to correlate with postoperative activity, although this could be the result of a beta error [18]. Furthermore, our available data showed no difference in long-term sports activity, whether patients were practicing sports before diagnosis or not.

The UCLA activity score reported a reduction in sports activity levels from 9 to 6 points at 3 years compared with preoperative activity and remained relatively stable thereafter. This level of 6 (“regularly participating in moderate activities”) after 3 years appears similar to that reported after 6 years after elective THA [26, 33]. One study showed that 43% of patients undergoing elective THA reached a UCLA score of over 7 one year postoperatively, which is described as “regularly participating in active events such as bicycling” and is supposed to be highly active. Approximately the same amount (38%) of our patients with megaprostheses ultimately reached this level 5 years postoperatively. One study even showed that patients with osteoarthritis improve their preoperative UCLA scores from 4.5 to approximately 6 after 1 year [35], whereas our patients treated for a tumor had UCLA scores of 9 before surgery and activity decreased to 3 one year postoperatively, which is seen as “sometimes participating in mild activities, such as walking, limited housework and limited shopping.” Interestingly, the patients reported a larger reduction in modified Weighted Activity Score (from 6 to 3 points) in the same time period. This is likely the result of the methodological “weighting” of different sports according to their impact and because of recommendations of their surgeons, which makes the loss of higher impact in sports or lowering of frequencies more obvious. The decrease from 6 to 3 median points reported by the modified Weighted Activity Score reflects on average moderate activity levels. According to the authors, a score of 9 and greater was defined as high activity [21]. In this current study none was performing any high-impact sport beyond 1 year from their procedure, although five of the patients reported participating in high-impact sports before surgery. We presume that there is a difference in expectations between patients after joint arthroplasty after osteoarthritis compared with patients receiving megaprosthetic reconstructions for bone tumors. Patients with osteoarthritis likely expect relief from preoperative pain and a relatively normal lifestyle, whereas patients with bone tumors appear to accept limitations and may be concerned about failure of their reconstruction so that they modify their activities. However, in terms of UCLA activity, six of 16 reported high activity (7–9 points) and in terms of modified Weighted Activity Score 3 of 16 reported high activity at long-term followup. This difference may lie in different frequencies, which have a decisive impact on modified Weighted Activity Score but not on UCLA. For example, patients with UCLA 7 “regularly participating in active events such as bicycling” can have higher or lower modified Weighted Activity Scores according to the frequency of bicycling. In terms of time and commitment in sport, seven of 16 patients after megaprostheses reached a mean of 3 hours/week sports activity, which is the recommended workout hours (4 hours/week) for healthy individuals [10].

Ollivier et al. reported that higher activity levels led to risk of implant failure and lower implant survivorship [25]. In this current study nine of 16 patients developed complications during their postoperative period and had revision surgery. Apart from a possible relationship of high activity and cup loosening (failure Type II) as well as mechanical disconnection of a module and loosening of the cone at another time (failure Type III), with the numbers of patients we had, we could not demonstrate an obvious sports-related association with prosthetic failures. Two patients were continuously practicing alpine skiing and did not shown negative effects on acetabular and femoral components so far, but with a larger number of patients and longer followup, it may be shown that a correlation does exist [12]. Similarly, the patients who took up playing golf after hip arthroplasty may also not lead to higher revision rates after hip arthroplasty, but we do not have a sufficient number of patients playing golf to know if the same is true for patients with tumor [19].

Our data can show that patients after tumor megaprostheses of the proximal femur can regularly play moderate sports and that long time periods are required to recover and adjust after operations of this magnitude but can reach comparable levels to patients after elective hip surgery after 5 years. However, the fact that patients can participate in sports with these implants does not mean that they should do so. Future studies will need to evaluate carefully–in the context of more complete, longer term followup of larger groups of patients–the degree to which sports participation might influence implant durability. With the numbers we had, we could not identify patient-specific factors predicting postoperative activity nor a relationship between type of surgery, implant, or surgical factors and activity levels. Sport activities may be an important part of some young persons’ lives, especially in a world of growing emphasis on mobility. Endoprosthetic implants for elective joint surgery attempt to adapt these requirements. Our data in this young cohort of patients suggest that there is a desire to participate in sports activities as reflected in the high number of patients participating in sports at 5-year followup, but this may not be true for all populations, cultures, or specific patients. With the numbers of patients we had, we could not show that sports was related to revision in survivors; however, failures resulting from higher activity levels with further followup cannot be excluded.
